# Rapid point-of-care CD4 testing at mobile units and linkage to HIV care: an evaluation of community-based mobile HIV testing services in South Africa

**DOI:** 10.1186/s12889-020-08643-3

**Published:** 2020-04-19

**Authors:** Rosa Sloot, Mary T. Glenshaw, Margaret van Niekerk, Sue-Ann Meehan

**Affiliations:** 1 Desmond Tutu TB Centre, Department of Paediatrics and Child Health, Faculty of Medicine and Health Sciences, Stellenbosch University, PO Box 241, Cape Town, 8000 South Africa; 2 Division of Global HIV/AIDS, Center for Global Health, Centers for Disease Control and Prevention, Pretoria, South Africa

**Keywords:** Linkage to care, Mobile HIV testing services (HTS), Point-of-care CD4 testing, South Africa

## Abstract

**Background:**

Mobile HIV testing services (HTS) are effective at reaching undiagnosed people living with HIV. However, linkage to HIV care from mobile HTS is often poor, ranging from 10 to 60%. Point-of-care (POC) CD4 testing has shown to increase retention in health facilities, but little evidence exists about their use in mobile HTS. This study assessed the feasibility of POC CD4 test implementation and investigated linkage to HIV care among clients accepting a POC test at community-based mobile HTS.

**Methods:**

This retrospective study used routinely collected data from clients who utilized community-based mobile HTS in the City of Cape Town Metropolitan district, South Africa between December 2014 and September 2016. A POC CD4 test was offered to all clients with an HIV positive diagnosis during this period, and a CD4 cell count was provided to clients accepting a POC CD4 test. Random effects logistic regression was used to assess factors associated with POC CD4 test uptake and self-reported linkage to care among clients accepting a POC test. Models were adjusted for sex, age, previous HIV test done, tuberculosis status and year of HIV diagnosis.

**Results:**

One thousand three hundred twenty-five of Thirty-nine thousand seven hundred ninety clients utilizing mobile HTS tested HIV positive (3%). 51% (679/1325) accepted a POC test. The age group with the highest proportion accepting a POC test was 50+ years (60%). Females were less likely to accept a POC test than males (odds ratio = 0.7, 95%CI = 0.6–0.8). Median CD4 count was 429 cells/μl (interquartile range = 290–584). Among 679 clients who accepted a POC CD4 test, 491 (72%) linked to HIV care. CD4 cell count was not associated with linkage to care.

**Conclusion:**

Our findings suggest that mobile HTS can identify early HIV infection, and show that a high proportion of clients with a POC test result linked to care. Future research should assess factors associated with POC test acceptance and assess the impact of POC CD4 testing in comparison to alternative strategies to engage HIV positive people in care.

## Background

South Africa has the largest burden of human immunodeficiency virus (HIV) worldwide, with 7.1 million people living with HIV in 2017 [1]. Early detection of HIV-infection and prompt receipt of antiretroviral therapy (ART) post diagnosis can optimize individual health benefit and prevent onward transmission [2–6]. Although these benefits are supported by strong evidence, they pose challenges for timely linkage to care, especially in high-burden, resource-constrained settings. In South Africa, similar to other sub-Saharan African countries, many people have failed to engage in care after HIV testing [7, 8]. In September 2016, South Africa implemented a universal test and treat policy, enabling free ART access for all HIV positive people regardless of CD4 count [9, 10]. However, in 2018, ART coverage remained at 62% among people living with HIV in South Africa [1].

Community-based HIV testing services (HTS) (conducted outside of a health facility) such as mobile testing units, contribute to early HIV detection as they have shown to reach previously undiagnosed, asymptomatic HIV positive people [11]. Mobile HTS units in South Africa have proven to achieve high coverage, facilitate testing of HIV positive people earlier in their disease course, and are more accessible to hard-to-reach populations, such as males, in comparison to stationary health facilities [12–15]. Although mobile units are successful for the delivery of HTS, subsequent linkage to care determines the effectiveness of mobile HTS units to improve patient outcomes and reduce population-level HIV transmission. Studies in South Africa investigating linkage to care show poor rates of linkage among populations diagnosed with HIV by mobile services, ranging from 10 to 60% [13, 16–18]. Linkage to HIV care can be challenging in community settings, where testers may be less symptomatic and unprepared for a new HIV diagnosis. Novel approaches are thus required to improve linkage to care at mobile units and thereby optimizing the full potential of mobile testing in community settings.

The use of point-of-care (POC) CD4 tests in stationary health facility settings, with provision of CD4 cell count test results at time of diagnosis, increases linkage to HIV care and may reduce loss to follow-up before ART initiation [19–22]. Few studies have reported on the utility of POC CD4 testing to reduce attrition between HIV testing and linkage to HIV care among people that self-initiated for HIV testing in a nonclinical approach, such as mobile HTS, in South Africa [23, 24]. Knowledge of CD4 cell count, combined with post-test counselling, might increase awareness of health implications and convince sceptical clients at mobile HTS units and motivate them to link to HIV care. We evaluated routinely collected data from clients who utilized mobile HTS units in five different communities in the Western Cape Province of South Africa from 2014 to 2016. In this study we (i) assessed the uptake of POC CD4 testing among HIV positive clients, (ii) quantified CD4 cell count to get an indication of disease progression among clients targeted by mobile HTS, and (iii) investigated the relationship between CD4 count level and linkage to HIV care among clients accepting a POC CD4 test.

## Methods

### Study setting

This retrospective cohort study used routine clinical records from clients who attended integrated community-based mobile HTS units between December 2014 and September 2016 in the City of Cape Town Metropolitan health district of the Western Cape Province of South Africa. The mobile HTS units were placed in communities as part of a service implementation project aimed to increase community-based HIV testing. This project was embedded within the health service system of the city of Cape Town and routinely collected data was provided monthly to relevant health services.

Mobile HTS units (including tents and mobile vans) provided HTS as described previously [15]. Mobile HTS units operated in five communities during the study period; one mobile unit was located in each community. All communities are characterised by low socio-economic status, and high HIV and tuberculosis (TB) disease burden [25–28]. Mobile HTS units were strategically placed at various locations within each community, such as transport hubs and along busy thoroughfares. Selection of these locations occurred on an ad hoc basis and changed regularly during the study period. Per South African national HIV testing guidelines [29], anyone aged 12 years or older could walk in without an appointment and request an HIV test. Healthcare workers (HCWs), including a nurse and at least two trained lay counsellors, worked a 40-h week at each mobile unit. Service delivery requirements determined the daily start and end of work times to accommodate flexi-time services and thereby reach clients who would otherwise not access the service. In addition to community-based HTS, other services included: TB symptom screening and testing, pregnancy testing and measurement of blood glucose, body mass index (BMI) and blood pressure. HCWs referred clients to public health facilities for further care as required.

HCWs asked clients about HIV testing history upon self-initiated attendance at mobile HTS. They did not complete an HIV test among clients who self-reported a positive HIV status, and they counselled self-reported positive clients who were not on ART to link to a public health facility for HIV care. HCWs took consent for an HIV test if the client never tested previously, or self-reported HIV negative or unknown HIV status. A client was diagnosed HIV positive if both the screening and confirmatory rapid test results were positive. In that case, the HIV test result was immediately given to the client. If the rapid screening test was positive, but the rapid confirmatory test was negative (discrepant result), blood was drawn and sent to the National Health Laboratory Service (NHLS) for an enzyme-linked immunosorbent assay (ELISA). A client with a discrepant result received appropriate counselling and was called by telephone when their ELISA result was available, approximately a week later, and was asked to return to the mobile unit to get their test result. Clients returning were counselled appropriately, depending on their test result. All clients diagnosed with HIV received a referral letter for HIV care at a public health facility of their choice.

POC CD4 testing was offered from December 2014 onwards at all five mobile HTS units for clients with an HIV positive diagnosis. A CD4 cell count was provided to clients accepting a POC CD4 test. HCWs collected a finger prick blood sample among HIV positive clients who accepted the CD4 test, and processed the sample on site using the Pima™ Analyzer (Alere). The same HCW included the test result on the Counselling and Testing form, and sent the client back to a lay counsellor for post-test counselling and explanation of the CD4 test result to the client. All HIV positive clients received a referral letter for HIV care, irrespective of CD4 count. Additionally, clients were encouraged to attend a public health facility to start ART, depending on ART eligibility according to the South African National Guidelines at that time. During our study, clients with CD4 count ≤500 cells/μl were eligible for ART [30]. In case of technical issues, a backup Pima™ Analyzer was available and a technician could be consulted.

A HCW contacted clients within 2 weeks after being diagnosed with HIV to assess linkage to care at a public health facility. HIV care at the public health facility included pre-ART services, where clients received counselling and were encouraged to return to the clinic after 6 months for a new CD4 count test, and provision of ART services. If the client had not linked to care, or if the HCW could not reach the client, at least two more attempts were made at various times of the day over a 3-month period after HIV diagnosis. This study considered clients to have linked to HIV care if they self-reported linkage to care at a public health facility for HIV care within 3 months after being diagnosed at a mobile testing unit. Clients who could not be contacted by phone to confirm whether they linked to care or not, were considered to not have linked to care.

### Data collection

This study used data from all clients who utilized the mobile HTS units between December 2014 and September 2016. Sex and age were recorded for each client as well as TB testing results, and whether clients had a previous HIV test done. HCWs routinely captured demographic and clinical data of each client on the counselling and testing (CT) forms. Client CT forms were kept at the HIV testing service for 3 months before being transported to a central data office. A Microsoft ACCESS 2013 database was specifically designed for this study. Two independent data clerks entered data into two separate datasets, after scanning the client’s unique barcode into the dataset. A third data clerk compared the datasets and validated any differences by referring to the source data (CT forms). This resulted in a final anonymized dataset, with no individual identifiers. All clients 12 years or older, who had an HIV test done and had a documented HIV test result available, were included in the analysis.

### Statistical analysis

Our study aims were to (i) describe characteristics of HIV positive clients diagnosed at mobile HTS, (ii) assess the uptake of POC CD4 testing, (iii) quantify CD4 cell count to get an indication of disease progression among clients targeted by mobile HTS, and (iv) investigate the relationship between CD4 cell count and linkage to HIV care among clients accepting a POC CD4 test. We used random effects logistic regression analysis to assess factors associated with acceptance of a POC CD4 test among clients who were offered a POC CD4 test, and to investigate the relationship between CD4 cell count and linkage to HIV care among clients accepting a POC test. The random effects approach specified community as the clustering variable (each mobile unit was located within a different community). We hypothesised that clients coinfected with TB were more likely to have symptoms which could influence POC CD4 test acceptance and linkage to HIV care. Similarly, clients previously tested for HIV might be more likely to engage in care. All models were therefore adjusted for TB status and previous HIV test done in addition to sex and age. Models were also adjusted for year of HIV diagnosis, to account for unknown time-dependent factors that could influence outcomes. We present unadjusted and adjusted odds ratios (ORs) and 95% confidence intervals (CIs) for each risk factor. We furthermore report median CD4 cell count among clients who accepted a POC CD4 test. Differences were assessed using the Chi-squared test, and for continuous variables the Mann-Whitney U test or Kruskal-Wallis test were used. Analyses were completed in Stata (StataCorp. 2015. Stata Statistical Software: Release 14. College Station, Texas, USA: StatCorp LP).

## Results

**Fig. 1:**
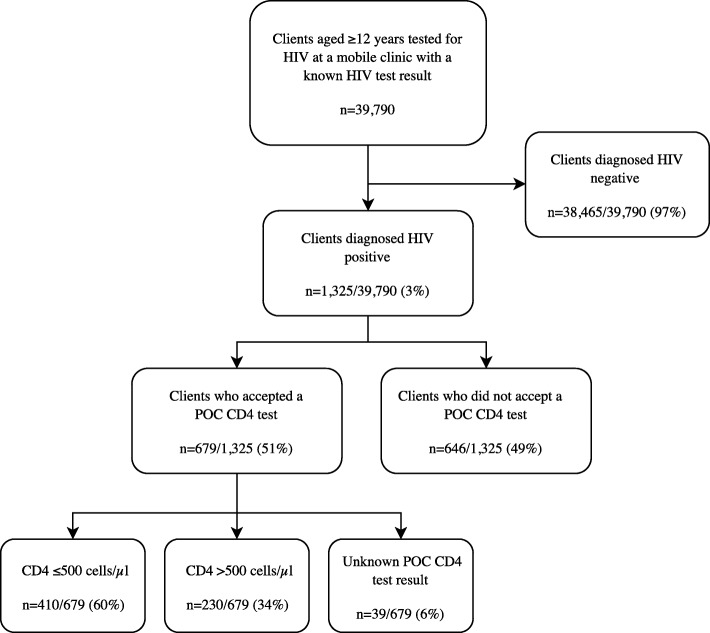
Chart of adolescents and adults tested for HIV at mobile HIV testing services in Cape Town, South Africa from 2014 to 2016. HIV = human immunodeficiency virus. POC = point-of-care

From December 2014 to September 2016, 39,790 clients aged 12 years or older were tested for HIV at mobile HTS units, of which 1325 (3%) tested positive for HIV (Fig. 1). Among 1325 HIV positive clients, 679 (51%) accepted POC testing. The majority of clients who had a POC test done had a CD4 cell count of ≤500 cells/μl (410/679, 60%).

Most clients were female (64%) and were aged 30–39 years (34%) Table 1. Clients in youngest (12–18 years) and oldest (50+ years) age groups were underrepresented at mobile HTS during 2014–2016 (both below 10%). Majority of clients had previously tested for HIV (87%), and TB coinfection was rare (< 1%) (Table 1).

**Table 1:** Characteristics of HIV positive clients diagnosed at mobile HIV testing services in Cape Town, South Africa from 2014 to 2016

	Study population ***n*** (%)
**Total**	1325
Sex	
**Male**	474 (36)
**Female**	851 (64)
Age (years)	
**Median (IQR)**	30 (24–37)
**12–18**	95 (7)
**19–24**	251 (19)
**25–29**	284 (21)
**30–39**	450 (34)
**40–49**	167 (13)
**50+**	77 (6)
**Unknown**	1
Previously tested for HIV	
**No**	175 (13)
**Yes**	1150 (87)
TB positive diagnosis	
**No**	1322 (100)
**Yes**	3 (0)

*HIV* human immunodeficiency virus, *TB* tuberculosis, *IQR* interquartile range

The lowest proportion of clients accepting a POC test was among clients aged 12–18 years (38%), and the highest proportion was found among clients aged 50+ years (60%) (Table 2). Females were less likely to accept POC CD4 testing than males (aOR = 0.7, 95%CI = 0.6–0.8). No other factors were associated with POC test acceptance.

**Table 2:** Factors associated with accepting a POC CD4 test among HIV positive clients diagnosed at mobile HIV testing services in Cape Town, South Africa from 2014 to 2016

	Accepted a POC CD4 test ***n*** (%)	Total ***n***	Unadjusted OR (95%CI)	***p***-value	Adjusted OR (95%CI)^**a**^	***p***-value
**Total**	679 (51)	1325				
Sex						
**Male**	272 (57)	474	1		1	
**Female**	407 (48)	851	0.7 (0.6–0.8)	< 0.001	0.7 (0.6–0.8)	< 0.001
Age (years)						
**12–18**	36 (38)	95	1		1	
**19–24**	117 (47)	251	1.4 (0.9–2.1)	0.055	1.4 (0.9–2.3)	0.110
**25–29**	145 (51)	284	1.7 (1.0–2.9)	0.040	1.6 (0.9–2.7)	0.075
**30–39**	249 (55)	450	2.0 (1.1–3.8)	0.024	1.9 (0.9–3.6)	0.050
**40–49**	85 (51)	167	1.7 (1.1–2.7)	0.026	1.6 (0.9–2.5)	0.060
**50+**	46 (60)	77	2.4 (1.1–5.3)	0.027	2.1 (0.9–4.7)	0.076
**Unknown**	1	1				
Previously tested for HIV						
**No**	83 (47)	175	1		1	
**Yes**	596 (52)	1150	1.2 (0.8–1.8)	0.392	1.2 (0.8–1.7)	0.431
TB positive diagnosis						
**No**	677 (51)	1322	1		1	
**Yes**	2 (67)	3	1.9 (0.1–26.3)	0.630	1.5 (0.1–22.0)	0.749

*POC* point-of-care, *HIV* human immunodeficiency virus, *TB* tuberculosis, *OR* odds ratio, *CI* confidence interval

^a^Multivariable model was adjusted for sex, age, previously tested for HIV, TB diagnosis, and year of HIV diagnosis

Six hundred forty of the Six hundred seventy-nine HIV positive clients who accepted POC testing had a CD4 cell count result available and had a median CD4 count of 429 cells/μl (IQR = 290–584) (Table 3). Males had a significantly lower median CD4 count (370, IQR = 273–501) than females (464, IQR = 297–641) (*p* < 0.001). Age group 12–18 years had the highest median CD4 count (565, IQR = 445–764), and the lowest median CD4 count was found among age group 30–39 years (392, IQR = 253–552) (Table 3).

**Table 3:** Median CD4 cell count of HIV positive clients who had a POC CD4 test result available at mobile HIV testing services in Cape Town, South Africa from 2014 to 2016

	POC CD4 test done	Median CD4 cell count (IQR)	***p***-value*
**Total**	640	429 (290–584)	
Sex			
**Males**	258	370 (273–501)	< 0.001
**Females**	382	464 (297–641)	
Age (years)			
**12–18**	36	565 (445–764)	< 0.001**
**19–24**	109	452 (327–617)	
**25–29**	135	454 (354–589)	
**30–39**	235	392 (253–552)	
**40–49**	78	431 (254–586)	
**50+**	46	422 (255–532)	
Previously tested for HIV			
**No**	80	352 (246–565)	0.047
**Yes**	560	438 (294–586)	
TB positive diagnosis			
**No**	638	432 (290–585)	0.029
**Yes**	2	118 (50–186)	

*POC* point-of-care, *IQR* interquartile range, *HIV* human immunodeficiency virus, *TB* tuberculosis

* Mann–Whitney U-test, unless stated otherwise

** Kruskall-wallis test

Among 679 clients who accepted a POC CD4 test, 491 (72%) linked to HIV care (Table 4). Linkage to care among clients not accepting a POC test was lower (375/646, 58%) (results not shown). Clients in age group 50+ years were significantly more likely to link to HIV care than clients in age group 12–18 years in univariable analysis (OR = 2.4, 95%CI = 1.3–4.5), but not in multivariable analysis (OR = 1.8, 95%CI = 0.6–5.8). There was no significant difference in linkage to HIV care among clients with CD4 cell count > 500 cells/μl compared to clients with CD4 cell count ≤500 cells/μl, in both univariable (OR = 1.1, 95%CI = 0.7–1.7) and multivariable analysis (OR = 1.0, 95%CI = 0.7–1.6).

**Table 4:** Factors associated with linkage to HIV care among HIV positive clients that accepted a POC CD4 test between 2014 and 2016 at mobile HIV testing services in Cape Town, South Africa

	Linked to HIV care ***n*** (%)	Total ***n***	Unadjusted OR (95%CI)	***p***-value	Adjusted OR (95%CI)^*^	***p***-value
**Total**	491 (72)	679				
Sex						
**Male**	188 (69)	272	1		1	
**Female**	303 (74)	407	1.3 (0.9–1.9)	0.189	1.4 (0.9–2.1)	0.070
Age (years)						
**12–18**	24 (67)	36	1		1	
**19–24**	76 (65)	117	0.9 (0.5–1.6)	0.796	0.6 (0.3–1.3)	0.193
**25–29**	107 (74)	145	1.4 (0.7–2.9)	0.375	1.0 (0.4–2.6)	0.943
**30–39**	177 (71)	249	1.2 (0.7–2.3)	0.508	0.9 (0.4–1.9)	0.761
**40–49**	68 (80)	85	2.0 (0.9–4.3)	0.079	1.3 (0.4–4.5)	0.634
**50+**	38 (83)	46	2.4 (1.3–4.5)	0.008	1.8 (0.6–5.8)	0.305
**Unknown**	1	1				
Previously tested for HIV						
**No**	53 (64)	83	1		1	
**Yes**	438 (73)	596	1.6 (0.7–3.3)	0.238	1.6 (0.8–2.9)	0.154
TB positive diagnosis						
**No**	490 (72)	677	1		1	
**Yes**	1 (50)	2	0.4 (0.01–11.1)	0.576	0.3 (0.01–8.9)	0.475
CD4 count						
**CD4 ≤ 500 cells/μl**	295 (72)	410	1		1	
**CD4 > 500 cells/μl**	170 (74)	230	1.1 (0.7–1.7)	0.651	1.0 (0.7–1.6)	0.933
**Unknown**	26 (67)	39				

*POC* point-of-care, *HIV* human immunodeficiency virus, *TB* tuberculosis, *OR* odds ratio, *CI* confidence interval

* Multivariable model was adjusted for sex, age, previously tested for HIV, TB diagnosis, CD4 count, and year of HIV diagnosis

## Discussion

To achieve targets for HIV treatment and prevention, it is essential to identify effective approaches to engage HIV positive people in care. Mobile HTS are effective at reaching undiagnosed people living with HIV but linkage to HIV care from mobile HTS is often poor [13, 15–18], calling for innovative strategies to increase engagement in care. This study evaluated POC CD4 test uptake and self-reported linkage to HIV care following referral from community-based mobile HTS units in the Western Cape Province of South Africa among clients newly diagnosed with HIV who were offered a POC CD4 test. Uptake of POC CD4 testing at mobile HTS was less than optimal, but linkage to HIV care among clients accepting a POC test was relatively high compared to linkage to care from mobile HTS reported in previous studies. Results furthermore show that mobile units reached HIV clients with early disease, and that CD4 cell count did not influence linkage to HIV care.

Almost half (49%) of clients who were offered a POC CD4 test, did not accept the test. Other studies offering POC CD4 at mobile clinics reported lower [13] and higher [23] test acceptance. In our study, clients already had to wait approximately 20 mins for each of the screening and confirmatory HIV test results. Mobile testers are generally less symptomatic, thus, it could be that they were less motivated to have blood taken and wait another 20 mins for the POC CD4 test result. We found that females were less likely to accept a POC test. Future studies should assess factors associated with POC test acceptance, and identify strategies to increase acceptance among females.

Median CD4 cell count among clients who accepted POC testing in this study (429 cells/μl) was similar to other studies that conducted POC testing at mobile HTS units in South Africa, varying from 414 to 416 cells/μl [13, 23, 24]. These findings highlight the importance of expanding mobile HIV testing in comparable settings as these clients have the opportunity to start ART before symptoms develop, resulting in better treatment outcomes. This has especially become important with the increased asymptomatic population eligible for treatment following implementation of the universal test and treat policies. Previous studies in South Africa have shown that mobile HTS have the ability to increase access to HTS for males, who traditionally do not access HTS at clinics [12, 31]. Mobile services should therefore continue to be part of routine service delivery within primary health care.

A recent systematic review found that POC CD4 testing has substantially reduced attrition between CD4 testing and ART initiation when compared to conventional laboratory-based CD4 testing [21]. In this study 73% of clients with a POC CD4 test result linked to HIV care following referral from community-based mobile HTS. This is much higher than linkage to care among clients not accepting a POC test (58%) and rates reported in similar studies assessing linkage to care among HIV positive clients diagnosed by mobile services without POC CD4 test results available (10–60%) [13, 16–18]. We hypothesised that clients with lower CD4 count eligible for ART would be more likely to link to care than clients with higher CD4 count not eligible for ART. No association was found, suggesting that other factors might have contributed to the high linkage proportion found among clients accepting a POC test in this study. The additional client support and counselling received as part of POC CD4 testing might have increased awareness of the importance of HIV care, resulting in relatively high linkage rates compared to previous studies. However, we cannot exclude the possibility that clients accepting POC testing were more likely to have certain characteristics also associated with increased linkage to care. Only few studies have investigated the impact of introducing POC CD4 testing on linkage to care at mobile services in South Africa. A study by Larson et al., reported 62% of patients who accepted the POC CD4 test to have completed their referral visit within 8 weeks of HIV testing, compared to 47% of those not offered the POC CD4 test [23]. These findings suggest that POC CD4 testing can be an effective strategy to increase engagement in HIV care. Thus, despite CD4 testing no longer being part of the HIV test and treat guidelines, CD4 count measurement might still be useful to improve linkage to care from mobile clinics. However, another study conducted in South Africa found no improvement in self-reported linkage to care using POC technology compared to standard of care [24]. More studies are required to investigate whether POC technology at mobile HTS units creates the opportunity to alert a group that would otherwise not have enrolled in HIV care immediately.

The study has some limitations. First, we relied on self-reported linkage to HIV care. It was not possible to verify client’s statements against health facility records, which would have increased reliability of our findings. It might be that linkage to care in this study is underestimated, as some clients may have linked after the 3-month follow-up period, but this could have been balanced by over-reporting of socially desirable answers given over the phone. Two previous studies investigating linkage to care after HIV diagnosis at mobile units in South Africa used clinical records to confirm self-reports of its clients. While the one study found perfect agreement between self-reports and clinic records [32], the other study showed that self-reported linkage was higher than verified [24]. Second, only few variables were available to be evaluated because the study used routinely collected data. We can thus not exclude the possibility that other factors than those recorded in our study contributed to POC test acceptance and linkage to HIV care.

## Conclusions

Identifying effective approaches to engage HIV positive people in care is essential to optimise the benefits of free ART for all HIV positive people. Our study shows that mobile HTS is effective at targeting people early in disease and findings suggest that POC CD4 testing can be used as a strategy to assist linkage to HIV care. However, a large proportion did not accept the test, and almost 30% did not link despite a CD4 count result. Our findings add to the limited evidence of POC CD4 testing and linkage to HIV care from mobile units in South Africa. Experimental studies are required to determine the impact and cost-effectiveness of POC CD4 testing at mobile HTS in comparison to alternative strategies to engage HIV positive people in care. Mobile units are now widely implemented across South Africa, and more evidence is necessary to inform policy makers to make decisions about ways to improve linkage to care.

## Data Availability

The datasets used and/or analysed during the current study are available from the corresponding author on reasonable request.

## Abbreviations

HIV: Human immunodeficiency virus
ART: Antiretroviral therapy
TB: Tuberculosis
HTS: HIV testing services
POC: Point-of-care
HCW: Healthcare worker
BMI: Body mass index
NHLS: National Health Laboratory Service
ELISA: Enzyme-linked immunosorbent assay
CT: Counselling and testing
ORs: Odds ratios
CIs: Confidence intervals
IQR: Interquartile range

## References

[1] Joint United Nations Programme on HIV/AIDS (UNAIDS). AIDS Info. 2018. Available at: http://aidsinfo.unaids.org/. Accessed 13 Dec 2019.

[2] Mugavero MJ, Lin HY, Willig JH (2009). Missed visits and mortality in patients establishing initial outpatient HIV treatment. Clin Infect Dis.

[3] Johnson LF, Mossong J, Dorrington RE (2013). Life expectancies of south African adults starting antiretroviral treatment: collaborative analysis of cohort studies. PLoS Med.

[4] Siedner MJ, Ng CK, Bassett IV, Katz IT, Bangsberg DR, Tsai AC (2015). Trends in CD4 count at presentation to care and treatment initiation in sub-Saharan Africa, 2002-2013: a meta-analysis. Clin Infect Dis.

[5] Iwuji CC, Orne-Gliemann J, Larmarange J (2016). Uptake of home-based HIV testing, linkage to care, and community attitudes about ART in rural KwaZulu- Natal, South Africa: descriptive results from the first phase of the ANRS 12249 TasP cluster-randomised trial. PLoS Med.

[6] The INSIGHT START Study Group (2015). Initiation of antiretroviral therapy in early asymptomatic HIV infection. N Engl J Med.

[7] Kranzer K, Govindasamy D, Ford N, Johnston V, Lawn SD (2012). Quantifying and addressing losses along the continuum of care for people living with HIV infection in sub-Saharan Africa: a systematic review. J Int AIDS Soc.

[8] Rosen S, Fox MP (2011). Retention in HIV care between testing and treatment in sub-Saharan Africa: a systematic review. PLoS Med.

[9] Department of Health RoSA (2016). Implementation of the universal test and treat strategy for HIV positive patients and differentiated Care for Stable Patients.

[10] Western Cape Government Department of Health. The Western Cape Consolidated Guidelines for HIV Treatment: Prevention of Mother- to- Child Transmission of HIV (PMTCT), Children, Adolescents and Adults. 2018. Available at: http://www.paediatrics.uct.ac.za/sites/default/files/image_tool/images/38/Western%20Cape%20Consolidated%20Guidelines%20for%20HIV%20Treatment%202018.pdf. Accessed 4 May 2019.

[11] Sharma M, Ying R, Tarr G, Barnabas R (2015). Systematic review and meta-analysis of community and facility-based HIV testing to address linkage to care gaps in sub-Saharan Africa. Nature.

[12] van Schaik N, Kranzer K, Wood R (2010). Earlier HIV diagnosis - are mobile services the answer?. S Afr Med J.

[13] Bassett IV, Regan S, Luthuli P (2014). Linkage to care following community- based mobile HIV testing compared with clinic-based testing in Umlazi township, Durban, South Africa. HIV Med.

[14] Bassett IV, Regan S, Mbonambi H (2015). Finding HIV in hard to reach populations: Mobile HIV testing and geospatial mapping in Umlazi township, Durban, South Africa. AIDS Behav.

[15] Meehan SA, Sloot R, Draper HR, Naidoo P, Burger R, Beyers N (2018). Factors associated with linkage to HIV care and TB treatment at community-based HIV testing services in Cape Town, South Africa. PLoS One.

[16] Labhardt ND, Motlomelo M, Cerutti B (2014). Home-based versus mobile clinic HIV testing and counseling in rural Lesotho: a cluster-randomized trial. PLoS Med.

[17] Maughan-Brown B, Harrison A, Galárraga O, et al. Factors affecting linkage to HIV care and ART initiation following referral for ART by a mobile health clinic in South Africa: evidence from a multimethod study. J Behav Med. 2019. 10.1007/s10865-018-0005-x. 10.1007/s10865-018-0005-x PMC6625943 30635862

[18] Dorward J, Mabuto T, Charalambous S, Fielding KL, Hoffman CJ (2017). Factors associated with poor linkage to HIV care in South Africa: secondary analysis of data from the Thol'impilo trial. J Acquir Immune Defic Syndr.

[19] Jani IV, Sitoe NE, Alfai ER (2011). Effect of point-of-care CD4 cell count tests on retention of patients and rates of antiretroviral therapy initiation in primary health clinics: an observational cohort study. Lancet.

[20] Patten GE, Wilkinson L, Conradie K (2013). Impact on ART initiation of point-of-care CD4 testing at HIV diagnosis among HIV-positive youth in Khayelitsha, South Africa. J Int AIDS Soc.

[21] Vojnov L, Markby J, Boeke C, Harris L, Ford N, Peter T (2016). POC CD4 testing improves linkage to HIV care and timeliness of ART initiation in a public health approach: a systematic review and meta-analysis. PLoS One.

[22] Desai MA, Okal DO, Rose CE (2017). Effect of point-of-care CD4 cell count results on linkage to care and antiretroviral initiation during a home-based HIV testing campaign: a non-blinded, cluster-randomised trial. Lancet HIV.

[23] Larson BA, Schnippel K, Ndibongo B (2012). Rapid point-of-care CD4 testing at mobile HIV testing sites to increase linkage to care: an evaluation of a pilot program in South Africa. J Acquir Immune Defic Syndr.

[24] Hoffmann CJ, Mabuto T, Ginindza S (2017). Strategies to accelerate HIV care and antiretroviral therapy initiation after HIV diagnosis: a randomized trial. J Acquir Immune Defic Syndr.

[25] Simbayi LC, Zuma K, Zungu N (2018). South African National HIV Prevalence, Incidence, Behaviour and Communication Survey, 2017.

[26] Statistics South Africa. Census 2011. Available at: http://www.statssa.gov.za/?page_id=3839. Accessed 4 May 2019.

[27] Western Cape Government Department of Health. Provincial Strategic Plan on HIV / AIDS, STIs and TB 2012–2016. Available at: https://www.westerncape.gov.za/assets/departments/health/provincial_strategic_plan_on_hiv_aids_stis_tb_2012_-_2016_-_15_june_2012.pdf. Accessed 4 May 2019.

[28] Wood R, Lawn SD, Caldwell J, Kaplan R, Middelkoop K, Bekker LG (2011). Burden of new and recurrent tuberculosis in a major south African city stratified by age and HIV-status. PLoS One.

[29] National department of Health, Republic of South Africa. National HIV Testing Services Policy 2016. Available at: https://sahivsoc.org/Files/HTS%20Policy%2028%20July%20final%20copy.pdf. Accessed 4 May 2019.

[30] South African National Department of Health (2015). National Consolidated Guidelines for the Prevention of Mother-To-Child Transmission of HIV (PMTCT) and the Managment of HIV in Children, Adolescents and Adults.

[31] Meehan SA, Naidoo P, Claassens MM, Lombard C, Beyers N (2014). Characteristics of clients who access mobile compared to clinic HIV counselling and testing services: a matched study from Cape Town, South Africa. BMC Health Serv Res.

[32] Govindasamy D, van Schaik N, Kranzer K, Wood R, Mathews C, Bekker LG (2011). Linkage to HIV care from a mobile testing unit in South Africa by different CD4 count strata. J Acquir Immune Defic Syndr.

